# Supplementary material for: Bi-directionality in translating culture: Understanding translator trainees’ actual and perceived behaviors
Source: PLoS One. 2023 Nov 15;18(11):e0293541. doi: 10.1371/journal.pone.0293541 (PMC10651051; doi:10.1371/journal.pone.0293541)
Supplement: S1 Appendix — (DOCX) [file pone.0293541.s001.docx]

**Appendix 1**

Text 1

Insiders describe their work in white house

The day is not composed of hours or minutes, but of news cycles. In each cycle, senior White House officials speaking on background define the line of the day. The line is echoed and amplified outside the beltway to real people, who live out there, by the President’s surrogates, whose appearances create actualities (on radio) and talking heads (on TV). During the roll-out of a new policy, the President coached by his handlers … may permit his own head to talk. There are various ways he might do this, ranging from the simplest photo-op to a one- on-one with a media big foot. (Guardian Weekend, 20 November 1993, original emphasis)

Questionnaire 1

Tick the strategy you have already used in translating the expressions below in the space

provided

| Strategies used in Translating the cultural references | | | | | | | | | |
| --- | --- | --- | --- | --- | --- | --- | --- | --- | --- |
|  | Identifying problems | Searching memory | Guessing | Contextualizing | Using search engines | Using a bilingual dictionary | Using a monolingual dictionary | Rereading | Revising |
| 1. *news cycles* |  |  |  |  |  |  |  |  |  |
| 1. on background |  |  |  |  |  |  |  |  |  |
| 1. line of the day |  |  |  |  |  |  |  |  |  |
| 1. the beltway |  |  |  |  |  |  |  |  |  |
| 1. actualities |  |  |  |  |  |  |  |  |  |
| 1. Talking heads |  |  |  |  |  |  |  |  |  |
| 1. Handlers |  |  |  |  |  |  |  |  |  |
| 1. Photo-op |  |  |  |  |  |  |  |  |  |
| 1. a one-on-one |  |  |  |  |  |  |  |  |  |
| 1. a media big foot |  |  |  |  |  |  |  |  |  |

Text 2

كانت متطلبات الدین الإسلامي ذات ضرورة لرصد دائم لقبة السماء الزرقاء، لأن الرسول صلى الله عليه وسلم

وضع قوانین ثابتة بفروض العبادة كما أن نزول عدد من الآیات في سور عدیدة تحدثت عن السماء والأفلاك والبروج والنجوم والأجرام السماویة والشمس والقمر، جعلت المسلم المؤمن یفكر بشأنھا، ویحترم ھذه القوانین ویحاول تطبیقھا. لذلك كان للمسلمین حاجة ماسة إلى علم النجوم لتعیین أوقات الصلاة وإثبات موعدي العیدین الأضحى والفطر، وتحدید سیر القوافل في الصحارى، وفي الملاحة البحریة في البحار والمحیطات، وكذلك معرفة أحوال الشفق وھلال رمضان – شھر الصوم – فكان یتطلب من الذي یرید إقامة الصلاة الاتجاه إلى القبلة في الكعبة بمدینة مكة المكرمة، وذلك یقتضي معرفة سمت القبلة، لأن زمن الصلاة یختلف حسب الموقع الجغرافي وحركة سیر الشمس في دائرة البروج، وكذلك معرفة أحوال الشفق والتماس ھلال شھر رمضان كل ذلك تطلب حل مسألة من مسائل علم الھیئة الكروي المبني على حساب المثلثات، وكذلك شروط رؤیة ھلال رمضان وأحوال الشفق للصوم والإمساك والإفطار، وكذلك صلاتا الكسوف والخسوف اللذین تتطلب معرفتھما استعمال الجداول الفلكیة

Questionniare 2

| Strategies used in Translating the cultural references | | | | | | | | | |
| --- | --- | --- | --- | --- | --- | --- | --- | --- | --- |
|  | Identifying problems | Searching memory | Guessing | Contextualizing | Using search engines | Using a bilingual dictionary | Using a monolingual dictionary | Rereading | Revising |
| 1. السماء الزرقاء |  |  |  |  |  |  |  |  |  |
| 1. الرسول صلى الله عليه وسلم |  |  |  |  |  |  |  |  |  |
| 1. عيد الأضحي |  |  |  |  |  |  |  |  |  |
| 1. عيد الفطر المبارك |  |  |  |  |  |  |  |  |  |
| 1. وھلال رمضان – |  |  |  |  |  |  |  |  |  |
| 1. إقامة الصلاة |  |  |  |  |  |  |  |  |  |
| 1. الاتجاه إلى القبلة |  |  |  |  |  |  |  |  |  |
| 1. حساب المثلثات |  |  |  |  |  |  |  |  |  |
| 1. صلاة الكسوف |  |  |  |  |  |  |  |  |  |
| 1. صلاة الخسوف |  |  |  |  |  |  |  |  |  |
