# Supplementary material for: Bi-directionality in translating culture: Understanding translator trainees’ actual and perceived behaviors
Source: PLoS One. 2023 Nov 15;18(11):e0293541. doi: 10.1371/journal.pone.0293541 (PMC10651051; doi:10.1371/journal.pone.0293541)
Supplement: Attachment — Submitted filename: Responses to Plos.docx [file pone.0293541.s003.docx]

Dear Editor of Plos One.

I hope this email finds you well. I am writing to submit the revised version of our manuscript titled " Bi-directionality in translating culture: Understanding translator trainees’ actual and perceived behaviors" following the invaluable feedback from the reviewers. We sincerely appreciate the reviewers' time and effort in providing constructive comments, which have significantly contributed to improving the quality of our work. Please find below our responses to each of the reviewers' comments. See the responses in the attachment.

Best Regards

- **Reviewer1**

| Reviewer’s comments | Response |
| --- | --- |
| Several in-text citations warrant careful verification to ensure alignment with the reference list. Additionally, one table lacks proper numbering, necessitating correction for coherence. Language-related issues have been identified in the conclusion and Text 2, affecting the overall clarity and fluency. | We've considered Reviewer 1's feedback. First, we've worked on improving the manuscript's clarity and fluency. We have enhanced the manuscript's language and fluency with the assistance of a native speaker. Additionally, we have reviewed and revised in-text citations with missing references to align them with the journal's guidelines. |

**Reviewer2**

| Reviewer’s comments | Response |
| --- | --- |
| What is the research background of this article? What was the specific study design? 2) Please add specific statistical analysis methods. 3) The results section should list all key values, not just a lack of evidence in language | All of these points were added in the revised version |
| 1. Introduction Paragraph 1, page 2: What are SL and TL? 1.2 Computational Model of Human Translation: I don't quite understand what you're trying to say in this paragraph. First of all, if it is to explain how Translog software records the behavior of students by tracking the behavior data of translators to illustrate the feasibility of this method, I think it is more appropriate to put it in the method part. Second, "But can translator trainees use these resources well? It depends on the search skills of the translator and the availability of information on the web...” What is the point of this paragraph? 1.3 Theoretical Framework: It is recommended to summarize and simplify the content. | Addressed and modified |
| Method: Note the serial number. | Addressed |
| Participants: There were 16 subjects in the study? Is the sample representative enough? I don't think your sample size meets the requirements. If yes, please provide corresponding theoretical support. And please improve the inclusion and exclusion criteria of study subjects. | The sample size was limited due to the utilization of Translog. To ensure students' familiarity with the software and its functionalities, tutorial sessions were necessary. It's important to note that participation in the study was voluntary, depending on the willingness of the participants. Furthermore, the process of extracting data from Translog is time-consuming, requiring manual retrieval of data for each individual participant. Some prior studies also employed a small sample size, for instance, Mohsen and Qassem (2019) with an N of 8 in their study "Analyses of L2 Learners’ Text Writing Strategy: A Process-Oriented Perspective" published in the Journal of Psycholinguistic Research by Springer. Chan S. (2017) utilized only 2 participants in "Using Keystroke Logging to Understand Writers’ Processes on a Reading-into-Writing Test" in Language Testing in Asia, also by Springer. Additionally, Pavlović N and Jensen K. (2009) employed eye-tracking in "Translation Directionality. As for the inclusion and exclusion criteria, potential participants had to meet several prerequisites to ensure comparability in L2 proficiency and educational background:  1. They are in the last year of the undergraduate translation program  2. The Participants completed an English proficiency measure: Brown's Cloze test^[1]^( M=60, SD = 9.20, N=18).  3. They have already taken part in the keylogging experiment and were familiar with the procedures.  4. Each participant provided informed consent to participate in this study.  5. Their typing speed is assessed (M= 19.57, SD= 0.06 per minute) so that the translation process is not affected by the typing variable, |
| Informant consent: Please supplement the ethical review. | It is verbal. |
| Please pay attention to the error in writing even the basic title number. And, the order of the entire method section is very chaotic, please follow the research design approach. | Addressed in the revised version. The manuscript was edited a native speaker of English. |
| Please unify all table formats into a three-line table, paying special attention to Table 5 and Table 6. | Addressed. |
| . Discussion 1) Where is Appendix 3? 2) Please describe the strengths and limitations of this study, and how the article will help future research.  6 | Addressed in conclusion |
| . Conclusion It is recommended to simplify the content. | Addressed |
| Your charts and tables are not standardized enough, like a "draft". And the entire text is too verbose, please pay attention to extracting key information. 4) Refine and simplify your introduction, I really can't read everything in its entirety. 5) Note the text format and numbering of the article. | Addressed |

- **Reviewer3**

| Reviewers’ comments | Response |
| --- | --- |
| The analysis of the data with a small number of participants (n = 16) should be checked by statisticians. The small sample size may require the use of other statistical procedures, such as non-parametric statistics tests. | We refined the analysis using non-parametric text, Wilcoxon test, which is an equivalent for paired sample t-test in parametric test (Mellinger and Hanson (2017). Major revisions were made in the section of results |

1. , a general English proficiency measure (Brown, 1980, 1988).
